# Current and Prospective Applications of CRISPR-Cas12a in Pluricellular Organisms

**DOI:** 10.1007/s12033-022-00538-5

**Published:** 2022-08-08

**Authors:** Shaheen Khan, Erwan Sallard

**Affiliations:** 1 Department of Molecular Biotechnology and Bioinformatics, Università degli Studi di Milano, Milan, Italy; 2 Division of Neuroscience, Department of Pharmacology & Toxicology, Vita-Salute San Raffaele University and Hospital, Milan, Italy; 3 Center for Biomedical Education and Research (ZBAF), Department of Human Medicine, Faculty of Health, Institute for Virology and Microbiology, Witten/Herdecke University, 58453 Witten, Germany

**Keywords:** CRISPR-Cas12a, Cpf1, Genome editing, Multiplex gene editing, Gene therapy, Plant biotechnology

## Abstract

CRISPR-Cas systems play a critical role in the prokaryotic adaptive immunity against mobile genetic elements, such as phages and foreign plasmids. In the last decade, Cas9 has been established as a powerful and versatile gene editing tool. In its wake, the novel RNA-guided endonuclease system CRISPR-Cas12a is transforming biological research due to its unique properties, such as its high specificity or its ability to target T-rich motifs, to induce staggered double-strand breaks and to process RNA arrays. Meanwhile, there is an increasing need for efficient and safe gene activation, repression or editing in pluricellular organisms for crop improvement, gene therapy, research model development, and other goals. In this article, we review CRISPR-Cas12a applications in pluricellular organisms and discuss how the challenges characteristic of these complex models, such as vectorization or temperature variations in ectothermic species, can be overcome.

**Supplementary Information:**

The online version contains supplementary material available at 10.1007/s12033-022-00538-5.

## Introduction

Clustered regularly interspaced palindromic repeats (CRISPR) are loci present in nearly all archea and half of bacteria species [1]. They are composed of repeats and spacers, the latter being inherited from phages or foreign plasmids. A spacer with part of the surrounding repeat sequences can be transcribed under the control of infection-induced regulators such as LeuO [2] and processed into a CRISPR RNA (crRNA) able to assemble with CRISPR-associated (Cas) proteins. Cas proteins have an endonuclease activity (generally DNase) and are subdivided in six types. Types I, III, and IV correspond to multi-subunit effector complexes, while types II, V, and VI gather single-subunit effectors [1, 3].

In prokaryotes, CRISPR systems play the role of adaptive immune systems against bacteriophage infections. A segment of the crRNA termed guide RNA (gRNA), transcribed from a CRISPR spacer, hybridizes to its complementary sequence on the target nucleic acid, which is then cleaved by the Cas nuclease. The cleavage is performed only if the target sequence includes a protospacer-adjacent motif (PAM), which is necessary for the binding of the Cas protein on DNA and the hybridization of the crRNA.

SpCas9 was the first Cas nuclease to be used artificially in 2012 [4]. More recently, other Cas proteins have been adapted for artificial use, including non-Cas9 proteins [5]. Different CRISPR systems have been optimized for a wide range of applications spanning from base editing to gene editing, silencing, or activation. They have been used in vitro, in bacteria, or in cell cultures, but also in pluricellular organisms [6]. The use of CRISPR systems in plants and animals may facilitate crop or farm animal improvement and developments in medicine. Nevertheless, it raises unique challenges such as vectorization, species particularities in genome accessibility or repair, or cell-type diversity [6, 7].

Cas12a or Cpf1 (CRISPR from *Prevotella* and *Francisella*), is a type V-A Cas protein present in a few dozen bacteria species [8]. In 2015, a screen of 16 Cas12a proteins revealed that Cas12a systems are suited for gene editing and that the Cas proteins of *Acidaminococcus sp*. (AsCas12a, or AsCpf1) and *Lachnospiraceae bacterium* (LbCas12a or LbCpf1) are the most efficient in human cells [8]. Since then, Cas12a has been increasingly used artificially [9]. The most frequently used orthologs are LbCas12a, AsCas12a, and FnCas12a. In plant cells, numerous other Cas12a orthologs proved highly efficient for gene editing, such as MAD7 [10], TsCas12a, ErCas12a, or Mb2Cas12a [11].

Cas12a displays unique features, such as higher specificity and ability to process crRNA arrays, turning it into an attractive alternative to Cas9. In this article, we comprehensively review the applications of Cas12a in plant and animal living organisms and discuss for which purposes it is most suited and which challenges it will face in future developments.

### Technologies Based on CRISPR-Cas12a

In this section, we discuss how Cas12a DNase activity can be harnessed for gene editing and more, using examples from pluricellular organisms.

#### Repair of Double Strand Breaks Generated by Cas12a

As other Cas nucleases, Cas12a produces double-strand breaks (DSB) in DNA sequences complementary with its gRNA. These targeted DSBs can be harnessed for several applications of gene editing.

**Fig. 1:**
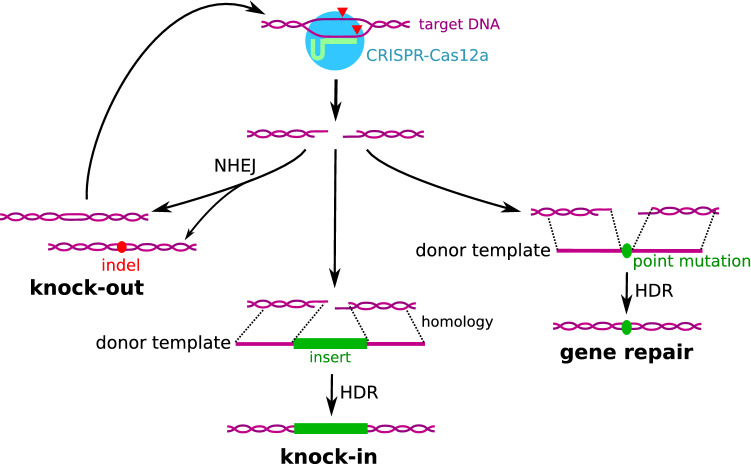
Repair mechanisms of Cas12a-induced DSB. Double-strand breaks generated by Cas12a in target cells can be repaired by non-homologous end joining (NHEJ) or homology-directed repair (HDR). NHEJ sometimes produces indels at the repair site and can thus be harnessed for knock-out applications. HDR can be harnessed to reverse mutations or insert transgenes depending on the donor co-delivered with the CRISPR-Cas12a system

1. *Knock-outs:* In higher eukaryotes, non-dividing haploid cells and eukaryotic cells in the G1 phase of the cell cycle, the main DNA repair pathway is non-homologous end joining (NHEJ) [12], a relatively error-prone pathway. Thus, a sequence targeted by CRISPR-Cas12a can be cut repeatedly by the nuclease until the repair mechanisms introduce indels (Fig. 1). When these mutations occur in exons, they can induce frame-shifts or less frequently insert stop codons or disrupt essential amino acids or splicing sites [7]. In each case, the protein produced by the targeted gene is likely to be inactivated or may not be synthesized due to non-sense mediated decay. Alternatively, introducing DSBs at two loci simultaneously can lead to the deletion of the sequences located between them. This process can be harnessed to knock-out kilobase to megabase-long sequences [10, 13].
2. *Knock-ins:* In certain cells, a DSB can be resolved through homology-directed repair (HDR). This requires a repair template with homology arms for both free DNA ends produced by the DSB. Consequently, CRISPR-Cas12a facilitates specific sequence integration when it is used alongside a donor template (Fig. 1). When the donor is a single-stranded DNA molecule, HDR is optimal when homology arms are around 80nt long [14], against 800 nt for double-stranded donor templates. To prevent new cleavage after the insertion, it is necessary to ensure that the crRNA is not able to bind the corrected locus. This can be achieved by ensuring that the PAM or part of the target sequence are deleted upon repair or by introducing synonymous mutations in the repair template [15].
3. *Gene repair:* HDR can also be harnessed in order to revert mutations, using a donor highly homologous to the targeted locus but carrying the wild-type allele instead of the mutation to be repaired (Fig. 1). Again, a usual method to prevent crRNA binding to the corrected sequence is to add synonymous mutations in the sequence complementary to the gRNA [15].

#### Multiplex Gene Editing

**Fig. 2:**
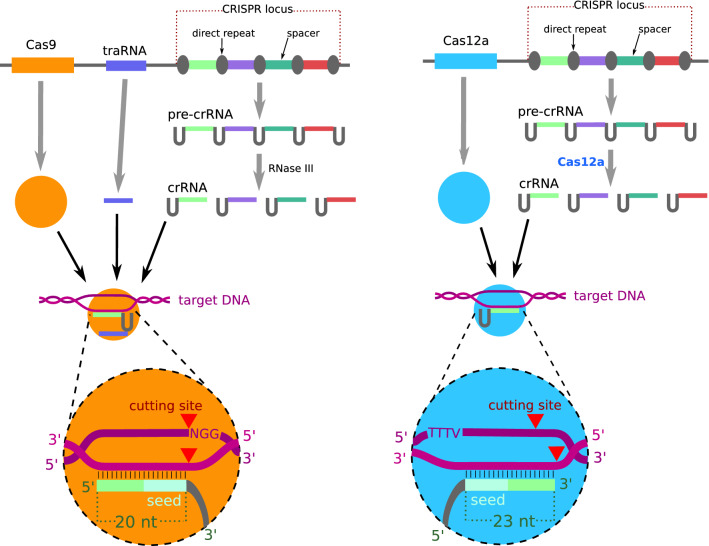
Expression, assembly, and function of CRISPR-Cas9 and CRISPR-Cas12a complexes. The protospacer-adjacent motifs (PAMs) indicated correspond to the most frequently used Cas9 and Cas12a nucleases, respectively, SpCas9 (NGG) and LbCas12a or AsCas12a (TTTV). The seed is the gRNA region where mismatches are least tolerated

Cas12a displays RNase activity and can thus process pre-crRNA transcripts into mature crRNAs [16] (Fig. 2). Consequently, a Cas12a protein and a crRNA array (i.e., a CRISPR locus analog containing multiple crRNAs in a single transcription unit) are sufficient to target and edit multiple sequences [17].

As a proof of concept of Cas12a multiplexing in mature animals, Zetsche et al. targeted three genes in mouse brains [17]. The targeted genes were mutated with efficiencies between 23 and 51%, and 17% of targeted cells carried mutations in the three genes simultaneously.

In rice, multiplex editing of up to 16 sites simultaneously achieved near 100% efficiency [11], using a crRNA array where crRNAs were flanked by hammerhead and hepatitis delta virus ribozymes. Indeed, ribozymes enhance crRNA processing in plants and enable to use a single Pol II promoter to drive both Cas12a and crRNA expression [18]. Pol II-driven crRNA expression is associated with more efficient gene editing in plants [19]. Similarly, the insertion of a tRNA sequence in 3′ of crRNA arrays resulted in crRNA stabilization and increased gene editing efficiency in mice [20] and rice [21].

#### Cas12a Beyond Gene Editing: CRISPRa and CRISPRi

Nuclease-dead variants of Cas12a cannot perform gene editing, but are still able to bind with high specificity the target sequence of their gRNA. Consequently, when Cas12a is fused with transcriptional activators or repressors, it can direct specific gene activation (CRISPRa) or silencing (CRISPRi).

Gene silencing with Cas12a has already been performed in *A*. *thaliana*: nuclease-dead AsCas12a fused with three SRDX transcriptional repressors decreased the expression of the target gene by more than 90% [19]. AsCas12a proved more efficient than LbCas12a for gene silencing even though it is less efficient for gene editing (Table 1) [19].

**Table 1:** Comparison of Cas12a orthologs

Cas12a variant	PAM	Temperature sensitivity	Currently tested organisms	Efficiency for gene editing	Efficiency for CRISPRa/CRISPRi
LbCas12a	TTTV	Intermediate	*A*. *thaliana*, wheat, rice, tobacco, soybean, cotton, tomato, citrus, drosophila, zebrafish, *Xenopus*, mouse, rat, human	Very high	High
AsCas12a	TTTV	Sensitive	*A*. *thaliana*, wheat, rice, tobacco, soybean, tomato, drosophila, zebrafish, *Xenopus*, mouse, rat, rabbit, pig	High	Very high
FnCas12a	TTN	Intermediate	Rice, silkworm	Variable	
Mb2Cas12a	TTV	Tolerant	Rice	Very high in rice	

Only the most prominent Cas12a orthologs are included in this table

PAM = protospacer-adjacent motif; V stands for A, G, or C, Y for C or T, and N for any nucleotide

For gene activation, a nuclease-dead LbCas12a fused to the VPR activator achieved a threefold increase in the expression of a Luciferase reporter gene in tobacco [22].

Multiplex gene silencing using nuclease-dead LbCas12a-SRDX was performed in rice and *A*. *thaliana* [11], with long-lasting silencing along two generations.

### Comparison of CRISPR-Cas12a and CRISPR-Cas9

Cas12a uses a single 42–44-nt-long crRNA with a 3′ terminal, 23–25-nt-long gRNA (Fig. 2) [8]. In comparison, SpCas9 and SaCas9, the most frequently used Cas9 variants, use respectively 20- and 21-nt-long gRNAs. The substantial length of the binding sequence may confer a higher target recognition specificity to Cas12a. Off-target activity is further reduced by the higher sensitivity of Cas12a to mismatches in the gRNA compared with Cas9 [23, 24].

DSBs generated by Cas12a expose staggered DNA ends with a 5′ overhang (Fig. 2) [25], which can lead to more efficient HDR repair than the blunt ends created by Cas9 [8, 26]. Furthermore, since Cas12a cutting site is distant from its PAM and seed sequence, NHEJ-induced mutations do not always prevent renewed binding and cleavage by Cas12a. This process increases the likelihood that the DSB is eventually repaired by HDR. Consequently, Cas12a appears better suited for knock-ins and gene repair than Cas9. This prediction was confirmed in zebrafish [27], rice [26], *A*. *thaliana* [15], tobacco [28], and tomato [29], where Cas12a induced knock-ins up to fourfold more efficiently than Cas9.

If no knock-in donor template is present, Cas12a target sites can be cut and repaired by NHEJ several times until a large deletion occurs and deletes the PAM, preventing further cleavage. Consequently, 3–30-nt deletions are frequently observed with Cas12a [10, 13, 30–33], whereas Cas9 mostly induces 1–2-nt indels [13]. Although this property may favor Cas12a for gene knock-outs [19, 33], preventing unwanted large deletions may be required for clinical use of Cas12a.

Cas12a requires 5′ terminal T-rich PAMs (Table 1, Fig. 2), whereas Cas9 uses G-rich PAMs. Consequently, these two nucleases can rarely target the same sequences. Depending on the nucleotide composition of the target locus, either nuclease can be more suited. Comparisons of Cas9 and Cas12a specificity and efficiency are thus challenging since these parameters heavily depend on the gRNA used, explaining contradicting results in different studies (Supplementary Table 1).

Recently, new variants of both Cas9 and Cas12a with other PAM requirements were developed [34–36]. Although they broaden the range of targetable sequences, Cas12a variants with modified PAMs have until now rarely been used in pluricellular organisms [11, 37].

Contrary to Cas9, Cas12a is able to perform collateral cleavage, that is sequence-unspecific cleavage of bystander single-stranded DNA or RNA molecules when the CRISPR-Cas complex is bound to its specific target [38]. This raised safety concerns for the use of CRISPR-Cas12a in vivo, but despite extensive genome-wide search no mutations produced by collateral DNA cleavage were detected in mice [39]. The apparent lack of bystander cleavage in living cells may be explained by the DNA repair mechanisms and the very low target concentration in vivo which decreases the probability of collateral cleavage.

The pre-crRNA of CRISPR-Cas9 are not processed by the Cas protein but by prokaryotic RNaseIII [40] (Fig. 2, Table 2). Thus, Cas9-based multiplex targeting requires one transcription unit for each crRNA or a cognate RNaseIII protein. Consequently, CRISPR-Cas12a renders multiplex targeting less complex and less technically challenging.

**Table 2:** Comparison of Cas12a with Cas9

property	Cas9	Cas12a
Protein length	SpCas9: 1368 amino acids, SaCas9: 1053 amino acids	1200–1500 amino acids (1228 for LbCas12a)
crRNA	2 in nature, 1 in synthetic applications (usually 101 nt long)	1 (usually 42 nt long)
gRNA	Around 20 nt	Around 23 nt
PAM	3′ terminal NGG (SpCas9)	5′ terminal TTTV (AsCas12a or LbCas12a)
Suited to target	G-rich regions	T-rich regions (such as promoters)
DNA cleavage	Blunt ends	Staggered ends with 5′ overhangs
RNAse activity	No	Yes
Collateral cleavage	No	Yes in vitro, not detected in vivo
Off-target activity	Low	Very low

Cas12a consistently achieved an efficiency comparable with Cas9 in pluricellular organisms [15, 26, 27, 29, 41, 42]. These two nucleases should be seen as complementary since each one of them outperforms the other one on certain target sequences and in certain systems and is more suited to particular applications (Table 2).

### Current Applications of Cas12a in Pluricellular Organisms

In this section, we take a closer look at the most significant applications of Cas12a already developed in pluricellular organisms. More information about studies describing Cas12a applications in pluricellular organisms is available in Supplementary Table 1.

#### Crop and Farm Animal Improvement

Cas12a is increasingly used for plant gene editing, both in model organisms and in crops, even though concrete applications are still relatively rare at the date of writing. Substantial efforts were made to optimize gene editing in technically challenging crops, such as soybean [43] or maize [44], with, for example, the use of high-activity nuclease variants.

In wheat, off-target frequency is around 10 times lower with LbCas12a and AsCas12a than with Cas9 [45]. To our knowledge, no off-target has yet been reported in other plants.

Current applications include the generation of *PGF* knock-out cotton plants whose grains may be edible [46], herbicide-resistant *A*. *thaliana* [15], salt-resistant tomatoes [29], and canker-resistant Duncan grapefruit [47], all using LbCas12a.

Multiplex editing is of particular interest in crops since agronomic improvements often depend on the editing of several genes. Therefore, Zhang et al. [11] successfully edited 6 sequences simultaneously to enhance yield and blight disease resistance of rice, taking advantage of the wider PAM requirements of Mb2Cas12a to edit sites that could not be targeted with LbCas12a or AsCas12a.

Finally, Cas12a-based gene editing has also been performed in farm animals. To counter nucleopolyhedrovirus (NPV) infections in *Bombyx mori* (silkworm) farms, Dong et al. created NPV-resistant transgenic silkworms expressing an anti-NPV crRNA and FnCas12a under a NPV-dependant promoter [41].

#### Disease Model Development

In rats and mice zygotes, Cas12a always displayed excellent specificity (only one off-target was reported [48] in nine studies (Supplementary Table 1)), low toxicity, and substantial efficiency (between 18.2 and 100% of genome-edited newborns were obtained from the zygotes) [20, 27, 39, 48–53]. Mutations were often successfully germline transmitted [48, 51]. Mosaicism was relatively rare in genome-edited rats (less than 28% of newborns) [51], but frequent in mice (up to 63%) [48, 49]. Mosaicism indicates that Cas12a frequently induces mutations after the one-cell stage of development and makes further breeding of genome-edited animals necessary to fixate the mutations. Mosaicism can be circumvented by performing the gene editing in cultured cells and transferring edited nuclei in embryos [20, 53].

Cas12a was used to generate several animal models for human diseases, such as atherosclerosis in rats (by multiplex knock-out of the *Apoe* and *Ldlr* genes) [51], Werner syndrome in rabbits [20], Duchenne muscular dystrophy (DMD) [20], and cryopyrin-associated periodic syndrome (CAPS) [53] in pigs.

#### Gene Therapy

An oncolytic adenovirus was armed with LbCas12a and a crRNA directed against the oncogene *EGFR* [54]. This vector was administered in mice tumors where it achieved a high gene knock-out efficiency (the EGFR protein concentration in the tumor was decreased by 92%) and inhibited tumor growth more than vectors without CRISPR systems. No off-target or cytotoxicity was detected.

Sun et al. knocked out the *PCSK9* gene in mouse hepatocytes [55] with 53% efficiency, leading to a twofold decrease in cholesterol levels. No cytotoxicity or off-targets were detected. This discovery paves the way for new treatments of cholesterol-related diseases in humans.

In a mouse model of macular degeneration, LbCas12a-mediated knock-out of *Vegfa* or *Hif1a* in retinal pigment epithelium cells prompted substantial therapeutic improvements [56]. Up to 17% of target cells were edited, by out-of-frame indels for 92% of them. No side effects were detected, off-targets were very rare (0.17% at most), and therapeutic efficiency was comparable to the standard-of-care. Consequently, Cas12a gene editing appears to hold great potential for safe long-term treatments of macular degeneration without the need of repetitive injections.

Cas12a-based gene therapy has already been used in humans: hematopoietic stem cells of thalassemia patients were edited ex vivo in order to knock out a pathogenic mutation creating a new splice site in the *HBB* gene [57]. 76.6% of treated cells displayed indels in the target sequence. Treated cells were reinjected to the patients, whose health significantly improved.

### Challenges of CRISPR-Cas12a Applications in Pluricellular Organisms

#### Cas12a Temperature Sensitivity

AsCas12a and to a lesser extent LbCas12a are temperature sensitive for gene editing. This hinders their applications in ectothermic species, such as drosophila [32, 33], zebrafish [27, 49], *Xenopus* [27], and plants. This issue can be circumvented by the use of temperature-tolerant orthologs, such as Mb2Cas12a (Table 1) [11], or temperature-tolerant AsCas12a [36] or LbCas12a [33] variants. For example, the D156R variant of LbCas12a achieved high gene editing efficiency in *A*. *thaliana* [58], tobacco [28], and drosophila [33] at temperatures as low as 22 °C.

In transgenic drosophila embryos expressing LbCas12a, gene editing can be induced at will in any developmental stage by temporarily increasing temperature from 18 to 29 °C [33]. This protocol increases the specificity of gene editing and helps to ensure that it occurs only in chosen organs. The temperature sensitivity of Cas12a is thus turned into an advantage.

Unlike gene editing, gene silencing by LbCas12a is temperature insensitive [59]. It suggests that unlike DNA cutting, DNA binding by LbCas12a is not increased by high temperatures.

#### CRISPR-Cas12a Delivery

Delivery of CRISPR-Cas12a requires optimized techniques, especially in pluricellular organisms where target cells are not directly available [7]. To counter this issue, Cas12a-based gene editing is often performed in single cells whose genome is easily accessible.

**Fig. 3:**
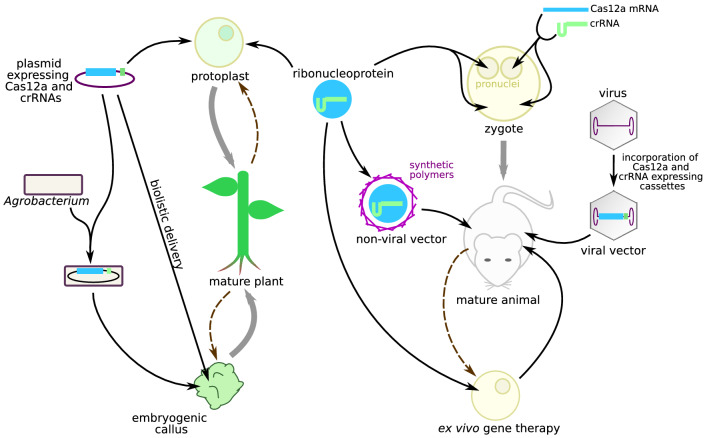
Main techniques of CRISPR-Cas12a vectorization. CRISPR-Cas12a systems can be delivered into mature organisms by viral or non-viral vectors. It is also possible to extract single cells or tissues, edit them, and reinject them in mature organisms or regenerate mature organisms from them. In these cases, CRISPR-Cas12a delivery can be performed by transformation, biolistics, or microinjection. In plants, transformation of calli and seedlings can be performed through *Agrobacterium* vectorization

In animal cells, for example, zygotes, the most frequent delivery techniques are 1-cytoplasmic or pronuclear microinjection of crRNA and Cas12a mRNA [48–51] and 2-electroporation or microinjection of Cas12a-crRNA ribonucleoproteins (RNPs) (Fig. 3) [27, 42, 52, 57]. Cas12a RNPs consistently proved more efficient than nucleic acid-based delivery methods, perhaps because crRNAs that are not protected in a RNP may be quickly degraded [27].

Due to the ability of plant cells to dedifferentiate, plants are often genome edited by isolating and dedifferentiating cells into protoplasts which can easily be transfected, edited, and selected, using the same delivery methods as for animal cells [60].

After genome edition, zygotes and protoplasts can regenerate pluricellular organisms. If the editing occurs at the one-cell stage, all cells of the organism will be edited. Gene editing of single cells is therefore adapted to crop or farm animal improvement and to numerous cases of disease model production.

For certain applications, the gene editing must be performed only in a specific cell type in mature pluricellular organisms: in this case, vectors are often necessary [7]. Vectors must be able to carry and protect or generate all components of CRISPR-Cas12a systems and to deliver them specifically to the target cells with high efficiency and safety. The natural ability of viruses or bacteria to infect their hosts can be harnessed through genome modifications that result in non-pathogenic vectors which deliver CRISPR-Cas12a components instead of bacterial plasmids or viral genomes (Fig. 3).

Adeno-associated viruses (AAV) and adenoviruses have already been used for CRISPR-Cas12a gene therapy in mice [54, 56], raising hopes of future clinical use in humans. Since the capacity of AAVs is limited to around 4.7 kb, the shorter size of Cas12a compared with SpCas9 is an advantage for vectorization. Furthermore, multiplex targeting is easily compatible with Cas12a vectorization, because Cas12a crRNAs are short and several crRNAs can be expressed in a single transcription unit. On the contrary, Cas9 requires multiple expression units, which make its use for multiplexing impractical.

Although viral vectors are highly efficient and cell type specific, they raise safety concerns due to potential immunogenicity, uncontrolled integration in host cell genome or reversal to pathogenic phenotypes, prompting the development of non-viral vectors. For example, Cas12a-crRNA RNPs can be delivered to target organs in PEI-coated DNA “nanoclews” carriers [55].

For applications in plants, plasmids encoding CRISPR-Cas12a can be vectorized in *Agrobacterium* to transform embryogenic calli or seedlings. Editing efficiency upon *Agrobacterium* transformation is higher than in protoplast editing, with up to 100% with LbCas12a in rice [37], 92% in soybean [61], and 90% in cotton [46]. However, transformation entails a higher degree of mosaicism [19, 31] than protoplast editing since the genome-edited organism is already pluricellular. Moreover, it can lead to the integration of CRISPR-Cas expressing cassettes in the plant genome [44].

Alternatively, RNPs or Cas- and crRNA-coding plasmids can be delivered into calli by biolistics [26, 44]. Finally, mature plants can be edited using plant viral vectors, with efficiencies of up to 88% [62].

Viral vectors and plasmid transformation can be combined. Van Vu et al. transformed tomato explants with geminivirus amplicons containing a donor template for repair, an LbCas12a gene, and two crRNAs [29]. The amplicons are delivered as plasmids, but in plant cells they can replicate as single-stranded DNA viruses [63]. As expected, the presence of single-stranded donor template increased HDR efficiency, with gene correction efficiency reaching 12.8%.

### Perspectives

In this article, we reviewed the applications of CRISPR-Cas12a in pluricellular organisms. Cas12a has already been used in numerous species and proved to be highly efficient and non-toxic in many situations. It outperformed Cas9 in specificity, knock-in efficiency, and multiplexing. LbCas12a is so far the most efficient and widely used Cas12a variant, particularly in plants, owing to its low temperature sensitivity. However, Mb2Cas12a may become the gold standard for gene editing of ectothermic organisms due to its apparent complete temperature tolerance.

Since Cas12a has been used for synthetic applications only since 2015 and in pluricellular organisms only since 2016, many developments are still underway. Notably, the patterns of sequence recognition and target cleavage are still partially unknown [16, 64]. A better understanding of these mechanisms may help to improve tools of CRISPR design.

Nuclease-dead Cas proteins fused with base editors can perform directed mutagenesis without the need of creating DSBs and repairing them through potentially mutagenic pathways [65]. Cas12a base editors may be applied in pluricellular organisms in a near future. However, Cas9 applications such as nicking or prime editing will be substantially more challenging to translate to Cas12a. Indeed, they require to cleave only one DNA strand, which is not possible with current Cas12a nucleases since they cleave both DNA strands with the same catalytic domain.

In plants, Cas12a may bring about substantial agronomic innovations. For example, LbCas12a facilitated directed crossing overs in maize in a proof-of-concept experiment [66], and this new technology may become prominent for crop hybridization and trait fixation.

LbCas12a has been used in primary human T cells in vitro with a multiplex system to simultaneously knock in anti-CD22 and anti-CD21 chimeric antigen receptor genes and knock out the immune checkpoint gene *PDCD1* [67]. The double knock-in reached 21.7% efficiency, that is 11-fold higher than with Cas9. This proof-of-concept study paves the way for Cas12a-based ex vivo gene therapies against leukemia that may reach the clinical stage in the coming years. Furthermore, this example illustrates that Cas12a holds great promises in oncology and gene therapy. Multiplexing could be used to treat multigenic disorders or tumors carrying several oncogenic mutations [54]. Even though no toxicity was reported in Cas12a-based gene therapy to date, broader clinical applications may require the development of low immunogenicity Cas12a variants [68].

## Supplementary Information

Below is the link to the electronic supplementary material.

Supplementary file1 (XLSX 16 KB)
